# The Legal Control of "Dispensaries" in New York

**Published:** 1898-01-22

**Authors:** 


### THE LEGAL CONTROL OF "DISPENSARIES" IN NEW YORK.

The New York *Medical News* gives the text of a Bill which it is proposed to submit at an early date to the Legislature in New York State with the object of bringing dispensaries under legal control. The conditions there are somewhat different from those with which we have to deal, in that many of the charities receive subsidies from the State, and that the State Board of Charities, by whom these subsidies are dispensed, forms a ready-made body naturally fitted for wielding the necessary powers. It will be noticed that the Bill provides for the issue and the withdrawal of licenses, without possessing which it shall be unlawful "for any dispensary to enter upon the execution or continue the prosecution of its purpose." Not only will it be unlawful to conduct an unlicensed dispensary, but it will "not be lawful to display or cause to be displayed in any manner whatsoever anything which could directly, or by suggestion, make public the existence of the equivalent, in purpose and effect, of a dispensary." As we read the Bill, however, it does not interfere with private rights. Any individual, at his own cost, may start and run a dispensary. A medical man may found a clinic without any fear. But he may not ask for subscriptions, nor may he even hope to get State help unless his dispensary has received its license. The Bill is, in fact, a measure to prevent subscriptions being collected, or trust funds and State subsidies being applied, for the support of any dispensaries which have not received the license of the State Board of Charities. A moderate measure of this kind ought to have a much better chance of becoming law than any more ambitious attempt to place the management of the dispensaries under State control. The great difficulty will be in regard to the action of the State Board of Charities. This Board has not shown any great willingness to discriminate even in the distribution of public funds. What amount of discrimination it will exercise in the distribution of licenses which cost nothing may well be doubted. Political party cries would be easily manufactured out of any action which looked like depriving a poor man of his physic! And political war cries are all powerful under a democracy. The text of the Bill is as follows:—

Section 1. By and for the purpose of this Act, a dispensary is declared to be any person, corporation, institution, society, association, or agent, whose purpose it is, either independently or in connection with any other purpose, to furnish, at any place or places, to persons non-resident therein, either gratuitously or for a compensation determined without reference to the value of the thing furnished, medical or surgical advice or treatment, medicine, or apparatus; provided, however, that the moneys used by and for the purposes of said dispensary shall be derived wholly or in part from trust funds, public moneys, or sources other than the individuals constituting said dispensary, and the persons actually engaged in the distribution of the charities of said dispensary.

Section 2. Six months after the passage of this Act it shall not be lawful for any dispensary to enter upon the execution, or continue the prosecution of its purpose unless duly licensed by the State Board of Charities as hereinafter provided.

Section 3. Upon the filing with the State Board of Charities of an application and statements in such form and of such substance as shall be prescribed by said Board, said Board may issue a license in such form as it shall prescribe to any dispensary, if in the judgment of said Board the statements filed and the evidence submitted therewith indicate that the operations of said dispensary will be for the public good.

Section 4. The State Board of Charities is hereby empowered to make rules and regulations, and to alter or amend the same, in accordance with which all dispensaries shall furnish, and applicants obtain, medical or surgical relief, advice or treatment, medicine or apparatus, but nothing in this Act contained shall be construed to mean that said Board shall have power to determine the particular school of medicine in accordance with which any dispensary shall manage or conduct its work, or to determine the kind of medical or surgical treatment provided by any dispensary.

Section 5. When it shall appear to the satisfaction of the State Board of Charities that any dispensary licensed by it under the provisions of this Act has violated any of the provisions of this Act, or any of the rules and regulations made by the said Board under authority of this Act, then, and in that event, the said Board is hereby empowered, due notice and an opportunity for a hearing having been given to said dispensary, to suspend or revoke the license of said dispensary.

Section 6. Six months after the passage of this Act no dispensary shall make use of any place commonly known as a drug-store, or any place or building defined by law or by an ordinance of a Board of Health as a tenement-house.

Section 7. Six months after the passage of this Act it shall not be lawful to display or cause to be displayed in any manner whatsoever anything which could directly, or by suggestion, make public the existence of the equivalent, in purpose and effect, of a dispensary.

Section 8. Any person who violates any of the provisions of this Act, or any of the rules and regulations made and published under the authority of this Act, shall be guilty of a misdemeanor, and, on conviction thereof, shall be punished by a fine of not less than ten dols., and not more than 250 dols.

Section 9. Nothing in this Act contained shall in any wise be construed to abridge any of the powers of the said State Board of Charities, or of any of the members or officers thereof, or inspectors duly appointed by it, now existing under and by virtue of Chapter 546 of the Laws of 1896, known as the State Charities Law.

Section 10. This Act shall take effect immediately.

